# Aurintricarboxylic Acid Decreases RNA Toxicity in a *C. elegans* Model of Repeat Expansions

**DOI:** 10.3390/toxins13120910

**Published:** 2021-12-20

**Authors:** Maya Braun, Shachar Shoshani, Anna Mellul-Shtern, Yuval Tabach

**Affiliations:** Department of Developmental Biology and Cancer Research, Institute for Medical Research Israel-Canada, Hebrew University of Jerusalem, Jerusalem 91120, Israel; maya.braun@mail.huji.ac.il (M.B.); shachar.shoshani@mail.huji.ac.il (S.S.); anmellul@gmail.com (A.M.-S.)

**Keywords:** CUG repeats, RNA interference, drug repositioning, *C. elegans*, small RNA pathway

## Abstract

Pathologic expansions of DNA nucleotide tandem repeats may generate toxic RNA that triggers disease phenotypes. RNA toxicity is the hallmark of multiple expansion repeat disorders, including myotonic dystrophy type 1 (DM1). To date, there are no available disease-modifying therapies for DM1. Our aim was to use drug repositioning to ameliorate the phenotype of affected individuals in a nematode model of DM1. As the RNA interference pathway plays a key role in mediating RNA toxicity, we investigated the effect of aurintricarboxylic acid. We demonstrated that by perturbing the RNA interference machinery using aurintricarboxylic acid, we could annihilate the RNA toxicity and ameliorate the phenotype. As our approach targets a universal disease mechanism, it is potentially relevant for more expansion repeat disorders.

## 1. Introduction

Pathogenic expansions of repeat sequences underlie over 40 neurodegenerative diseases, including myotonic dystrophy type 1 (DM1). DM1 is caused by expansions of the CTG repeats in the 3′ UTR of the *DMPK* gene. Healthy individuals typically carry 5–37 CTG repeats, while DM1 patients carry more than 50 repeats and may reach several thousand [1]. These expanded repeats trigger RNA toxicity that causes multisystemic symptoms. The RNAs transcribed from the expanded region were shown to disrupt cellular function through gain-of-function- or loss-of-function-type mechanisms. These include sequestration of RNA-binding proteins, such as the alternative splicing regulators MBNL1 and CUGBP1, microRNA and siRNA dysfunction, and RAN translation [2,3,4]. Due to its clinical and genetic variability, DM1 is difficult to treat and there is no disease-modifying therapy currently available [5].

In our previous work, we revealed the key role of the RNA interference (RNAi) machinery in mediating RNA toxicity in a *Caenorhabditis elegans* DM1 model [6,7]. The long RNAs transcribed from the expanded repeats are targeted by Dicer and processed to short-interfering RNAs (siRNAs). These siRNAs bind to Argonaute enzymes, recruit an RNA-induced silencing complex (RISC), and induce silencing of genes bearing complementary sequences [6,7,8]. In the human genome, endogenous 6 CTG repeats occur in over 900 genes, marking them as potential targets for RNAi-mediated silencing.

In this study, we investigated a drug-repurposing strategy, targeting the RNAi pathway for treating DM1. We focused on aurintricarboxylic acid (ATA), a triphenyl-methyl molecule that inhibits RNA binding to Argonaute and disrupts the assembly of the RISC [9].

## 2. Results

**Figure 1:**
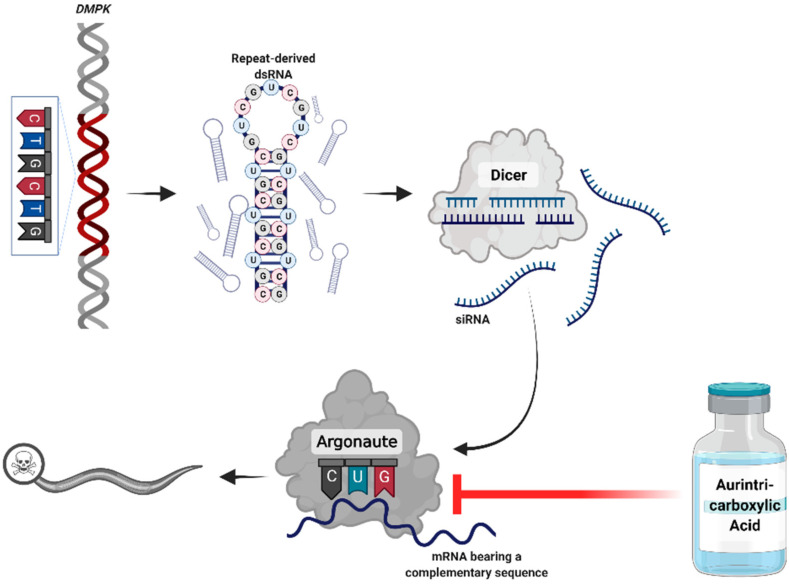
Proposed mechanism of the pathogenicity of repeat expansion disorders. RNA transcribed from the expanded DNA repeats creates hair-pin structures that accumulate in foci. These double-stranded RNAs are processed by Dicer to short-interfering RNA (siRNA). The siRNAs engage the downstream RNA interference components to silence genes bearing complementary sequences. Administration of aurintricarboxylic acid may disrupt this interaction. Created with BioRender.com (Accessed 3 November 2021).

The RNAi pathway plays a key role in pathogenesis of repeat expansion disorders (Figure 1) [6,7,8]. Accordingly, to identify drug repurposing candidates for this group of disorders, we focused on targeting the RNAi machinery. We searched the literature for drugs and compounds known to directly affect RNAi members or indirectly perturb the RNAi pathway. Then, we evaluated the results according to the safety profiles of drugs and the current uses for conventional indications. Based on the results, we decided to proceed with ATA for further investigation in an in vivo model.

**Figure 2:**
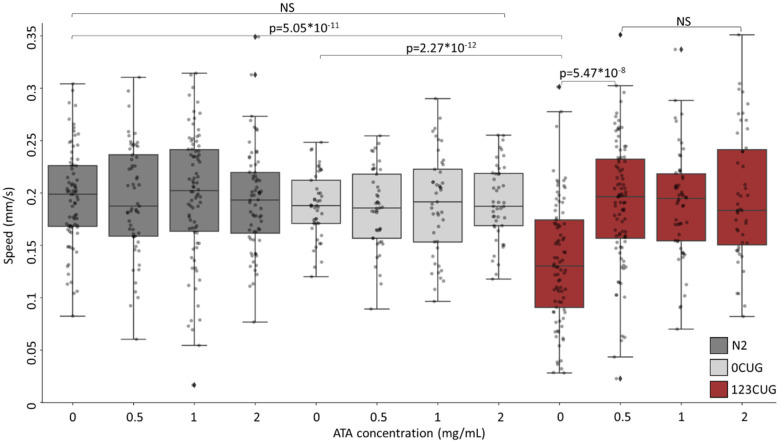
Administration of aurintricarboxylic acid to 123CUG nematodes rescues motility impairment. Motility assay (moving speed) of 2-day-old N2 (wild-type), 0CUG, and 123CUG nematodes (*n* = 60) following increasing concentrations of ATA treatment (0, 0.5, 1, and 2 mg/mL). Three biological replicates are represented. Significance was calculated using an ANOVA test following by post hoc two-tailed Student’s *t*-test.

We utilized a *C. elegans* experimental system of expanded repeats. The *C. elegans* strains express green fluorescent proteins (GFP) bearing 0 or 123 repeats of CTG (0CUG and 123CUG, respectively) in the 3′ UTR, under the regulation of the *myo-3* muscle-specific promotor [6,7,10]. Phenotypically, the toxicity induced by the expanded repeats manifests itself in motility impairment, expressed as slower crawling speeds in affected nematodes. Hence, we first examined the effect of ATA administration on nematode motility. We grew N2, 0CUG, and 123CUG nematodes on NGM plates with increasing doses of ATA (0 mg/mL, 0.5 mg/mL, 1 mg/mL, and 2 mg/mL). On day two of adulthood, we filmed the treated nematodes and measured their crawling speed (Figure 2). The 123CUG untreated nematodes exhibited severely impaired motility with an average speed of 0.14 mm/s as compared with the 0CUG and N2 groups, which both measured an average speed of 0.19 mm/s. Treatment of 0.5 mg/mL ATA achieved full rescue of motility impairment in 123CUG nematodes, with an average moving speed of 0.19 mm/s. The increased doses of 1 mg/mL and 2 mg/mL had the same effect. Importantly, ATA treatment did not affect motility in 0CUG and N2 control nematodes, in any of the studied concentrations.

**Figure 3:**
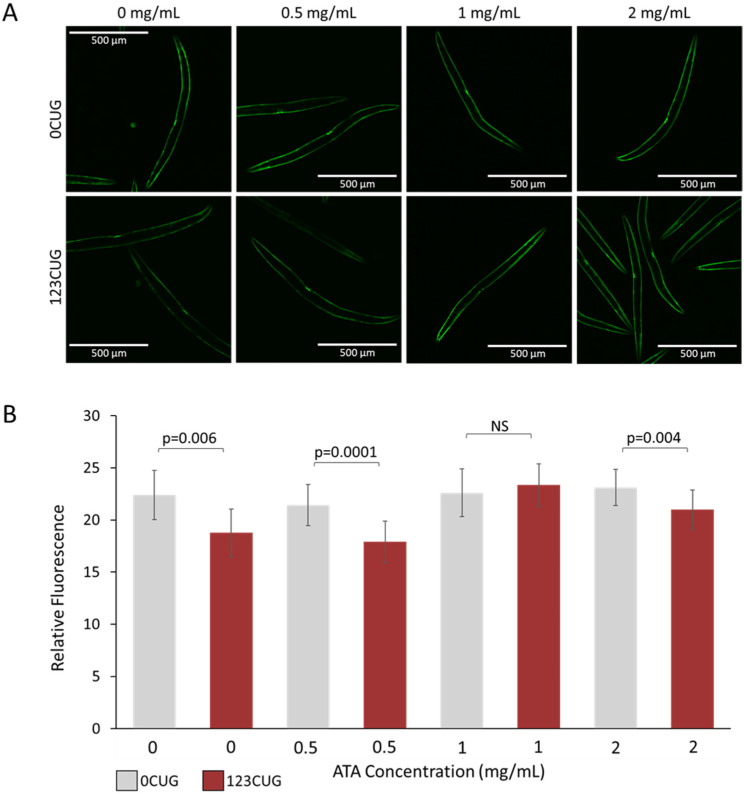
Administration of aurintricarboxylic acid (1 mg/mL) to 123CUG nematodes rescues fluorescence decay. Fluorescence assay of 2-day-old 0CUG and 123CUG nematodes (*n* = 30), following increasing concentrations of ATA treatment (0, 0.5, 1, and 2 mg/mL). Relative fluorescence was computationally quantified (**B**) and representative fluorescent microscopy images are shown (**A**). Data are represented as a mean ± SD of three biological replicates and significance was calculated using a two-tailed Student’s *t*-test.

An additional toxicity-associated phenotype observed in the 123CUG nematodes is characterized by an age-related decrease in GFP protein levels, compared with the 0CUG controls, observed as reduced fluorescence [6,10]. We have previously shown that downregulation of the RNAi pathway rescues GFP expression levels, suggesting that the decrease in fluorescence is due to siRNA-mediated silencing of the transgene. To establish whether ATA treatment rescues this phenotype, we measured and compared the fluorescence levels of 2-day-old adult 0CUG and 123CUG animals treated with increasing doses of ATA (0 mg/mL, 0.5 mg/mL, 1 mg/mL, and 2 mg/mL). Treatment of 1 mg/mL ATA achieved full rescue of the fluorescence decay in 123CUG animals (Figure 3). Note that the highest dose of 2 mg/mL had a significant rescue effect on the 123CUG nematodes; however, it was attenuated and did not reach the fluorescence levels of their matched controls, or those treated with lower doses.

**Figure 4:**
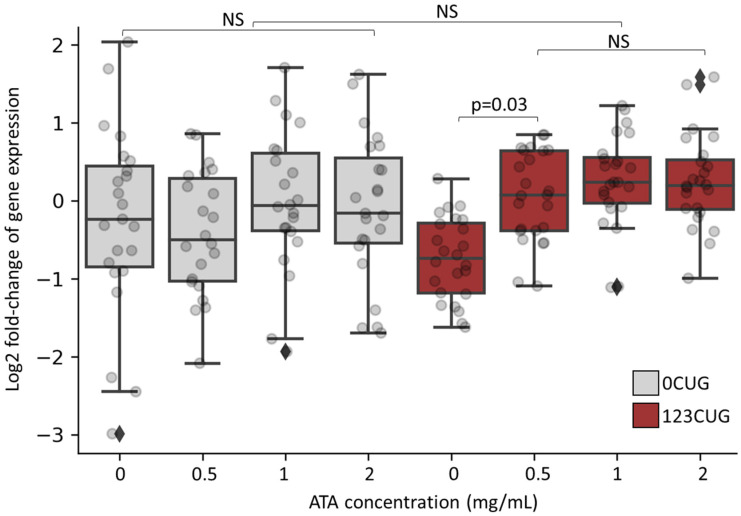
Increasing doses of aurintricarboxylic acid normalize expression levels of CTG-bearing genes in 123CUG nematodes. Log2 of gene expression fold change of 24 genes bearing ≥7 CTG/CAG repeats in 0CUG and 123CUG 2-day-old adults following treatment of ATA in increasing doses. Expression levels are normalized to untreated wild-type nematodes. The qPCR is an average of three biological experiments and three technical replicates.

As we previously showed, the repeat-derived siRNAs induce silencing of genes bearing complementary sequences through an RISC [6,7]. As ATA inhibits RNA binding to Argonaute and disrupts the assembly of the RISC, we anticipated a rescue of the gene silencing phenotype following its administration. Consequently, we measured the expression levels of genes bearing 7 CTG/CAG repeats, before and after ATA treatment. We collected RNA from 2-day-old adult 123CUG, 0CUG, and wild-type (WT) nematodes, following treatment with increasing concentrations of ATA. Using qPCR, we analyzed the expression of 24 genes containing 7 or more CTG/CAG repeats. Significant downregulation in expression was found in 2-day-old adult 123CUG nematodes as compared with 0CUG and WT nematodes (Figure 4 and Figure S2). ATA treatment, starting at the lowest dose of 0.5 mg/mL, rescued the reduced expression levels of these genes. The effect was also observed in higher doses.

Notably, there were no significant differences in lifespan between the WT and 123CUG groups following ATA treatment (Figure S1). However, the highest dose of 2 mg/mL slightly reduced the lifespan of the nematodes, as compared with the untreated groups’ lifespans, suggesting a toxic effect at high concentration.

## 3. Discussion

Our experiments showed that the administration of aurintricarboxylic acid reduced RNA toxicity in nematode models of repeat expansion disorders. The nematodes expressing expanded repeats (123CUG) mimicked disease phenotypes, such as impaired muscle function and alterations in expression levels of genes bearing complementary CTG sequences. We previously found that these phenotypes are manifestations of RNA toxicity, mediated by the RNAi machinery [6,7]. Dicer processes the RNA transcribed from the expanded repeats to short-interfering RNA [8]. Subsequently, the siRNAs recruited the RISC that targets genes with endogenous repeated CTG sequences and silences them. The chemical compound aurintricarboxylic acid (ATA) was reported to disrupt the assembly of a RISC [9].

We used a previously validated method to administer increasing doses of the compound to nematode growth medium (NGM) and found that they exert a significant biologic effect [11]. We found a toxicity signal at the highest dose. Physiological barriers, such as the nematodes’ impermeable cuticle and enzymatic detoxification system, affect the bioavailability of the drug at the target, therefore accounting for the higher drug concentrations in the culture required to achieve adequate bioactivity in live nematodes vs. mammalian cell cultures [9,12,13].

Following treatment with 0.5–2 mg/mL of ATA, the 123CUG nematodes exhibited vast improvement in motility and reached similar movement speed as the WT nematodes. Importantly, increasing the concentrations of ATA did not affect the speed of WT nematodes, implying no toxic effect on motility in the tested doses. Administration of ATA resolved the downregulation in expression levels of genes bearing 7CTG repeats in the 123CUG nematodes. The gene expression levels in the WT nematodes remained largely unchanged, excluding drug-induced toxicity (Figure S2). Note that the treatment of 123CUG nematodes successfully rescued the decay in fluorescence at a dose of 1 mg/mL, but the highest dose of 2 mg/mL failed to completely rescue this phenotype. Moreover, this dose reduced the lifespan of the 123CUG and wildtype nematodes. Taken together, we conclude that 0.5–1 mg/mL of ATA fulfills its therapeutic potential, while the dose of 2 mg/mL tends to be toxic. In conclusion, ATA treatment rescued disease phenotypes, such as muscle impairment, reduced fluorescence levels, and changes in gene expression.

Although ATA has various effects apart from disrupting RISC assembly, treatment of the control 0CUG and N2 nematodes did not affect the tested phenotypes. However, it successfully recapitulated the rescue effects we have previously achieved following direct manipulation of the RNAi pathway [6,7]. Moreover, the alterations in expression levels of genes bearing endogenous CTG repeats specifically indicate RNAi-mediated silencing, hence its rescue by ATA suggests perturbation of aberrant RNAi activity. Therefore, we believe that the main mechanism in which ATA reduces RNA toxicity in our model is through inhibition of the RNAi pathway. We suggest that, taken together, our findings prove the concept that using a drug described to inhibit RISC formation may ameliorate the disease phenotype in a model of nucleotide repeat disorders and may provide a framework to search for therapy in human disease. However, future work should assess the additional effects of ATA on this model, including inhibition of apoptosis, which may contribute to the impaired motility phenotype [14].

To date, repeat expansion disorders are managed symptomatically and there is no approved disease-modifying treatment. Although gene editing therapies are under development, they are not predicted to become available in the near future due to numerous obstacles [15]. Our results support further evaluation of ATA in mammalian models and investigation of additional compounds that may affect the RNAi pathway, as part of the pressing search for novel therapeutic approaches in expansion repeat disorders.

## 4. Materials and Methods

### 4.1. C. elegans Strains

*C. elegans* strains GR2024 (123CUG) and GR2025 (0CUG) were used [10]. The 123CUG or 0CUG repeats are expressed in the 3′ UTR of GFP in body wall muscle cells through the myo-3 promoter. The Caenorhabditis Genetics Center (Minneapolis, MI, USA) provided the N2 (Bristol) strain for use as a WT strain. Nematodes were handled using standard methods and grown at 20 °C, unless otherwise indicated.

### 4.2. Drug Administration

Aurintricarboxylic acid ammonium salt (A36883, Sigma-Aldrich, St. Louis, MI, USA) was dissolved in water to 10 mg/mL, as stock solution. The ATA stock solution was diluted with nematode growth medium (NGM) (below 65 °C after boiling) to final concentrations of 0.5 mg/mL, 1 mg/mL, and 2 mg/mL. The NGM-ATA was poured into petri plates and live *E. Coli* OP50 bacteria were seeded.

### 4.3. Motility Assay

Synchronized N2, 0CUG, and 123CUG *C. elegans* nematodes were grown on NGM plates containing increasing doses of ATA (0, 0.5, 1, 2 mg/mL) until day two of adulthood. Then, 5 *C. elegans* nematodes were picked and placed on 60 mm NGM plates without food. Following 20 min of recovery, the nematodes were filmed. The experiment was conducted with over 15 animals per replicate and the data from three biological replicates were combined. Digital microscope images were captured using MICAM 4.0 software. Sixty frames were taken at a rate of one capture per second. All images were captured at room temperature and with the same focus in each experiment. A playback rate of 15 frames per second was used to construct the video using MakeAVi software. Using Tracker 5.0, the tail of the animals was defined as a point mass, and the position of the tail was tracked for each frame. Statistical analysis was conducted with Student’s *t*-test, with α = 0.05.

### 4.4. Real-Time Quantitative Polymerase Chain Reaction (RT-qPCR) Analysis of Gene Expression

Hundreds of N2, 0CUG, and 123CUG nematodes were grown on NGM plates with different concentrations of aurintricarboxylic acid starting from the first larval stage. On day two of adulthood, nematodes were collected and washed using M9. Total RNA was isolated from *C. elegans* nematodes using a Trizol Reagent (Ambion, Austin, TX, USA) and a NucleoSpin RNA isolation kit (Macherey-Nagel, Duren, Germany). A kit for reverse transcription of cDNA (Applied Biosystems, Carlsbad, CA, USA) was used and the mRNA expression levels were determined by qPCR. The CFX-384 Real-Time PCR system (Bio-Rad, Hercules, CA, USA) was utilized with SYBR-Green (Bio-Rad, USA). Analyses were conducted using the ∆∆Ct method. Using *rpl-32* and *cdc-42* as reference genes, gene transcript quantities were calculated. All the primers used in this research were designed using the NCBI Primer Blast (sequences depicted in Table S1).

### 4.5. Fluorescence Assay

Synchronized 0CUG, and 123CUG C. elegans nematodes were grown on NGM plates with increasing doses of ATA (0, 0.5, 1, 2 mg/mL) until day two of adulthood. The animals were washed twice with M9, anesthetized using 10 mM sodium azide (Sigma Aldrich, St. Louis, MI, USA), and placed on an agar pad. Images were taken using a spinning disk confocal microscope. Within the same figure panel, images were collected with the same exposure settings and processed in ImageJ identically. Statistical analyses were performed using a two-tailed Student’s *t*-test, α = 0.05.

### 4.6. Lifespan

Synchronized eggs were placed on NGM-ATA plates seeded with OP50. The eggs were incubated at 20 °C until they reached the L4 larval stage, and 60 animals were transferred to fresh plates (10 worms per plate). Dead worms were scored every two days and transferred onto freshly seeded plates every four days.

## Supplementary Materials

The following are available online at https://www.mdpi.com/article/10.3390/toxins13120910/s1, Figure S1: Lifespan of N2 and 123CUG following treatment with increasing doses of ATA, Figure S2: Gene expression of CTG-bearing genes in N2 nematodes following treatment with increasing doses of ATA, Table S1: Primer sequences for RT-qPCR.
