# Supplementary material for: Aurintricarboxylic Acid Decreases RNA Toxicity in a C. elegans Model of Repeat Expansions
Source: Toxins (Basel). 2021 Dec 20;13(12):910. doi: 10.3390/toxins13120910 (PMC8706575; doi:10.3390/toxins13120910)
Supplement: Supplementary file 1 [file toxins-13-00910-s001.zip › toxins-1472678-supplementary.pdf]

Supplementary Materials: Aurintricarboxylic Acid Decreases RNA Toxicity in a *C. elegans* Model of Repeat Expansions

Maya Braun, Shachar Shoshani, Anna Mellul-Shtern, and Yuval Tabach

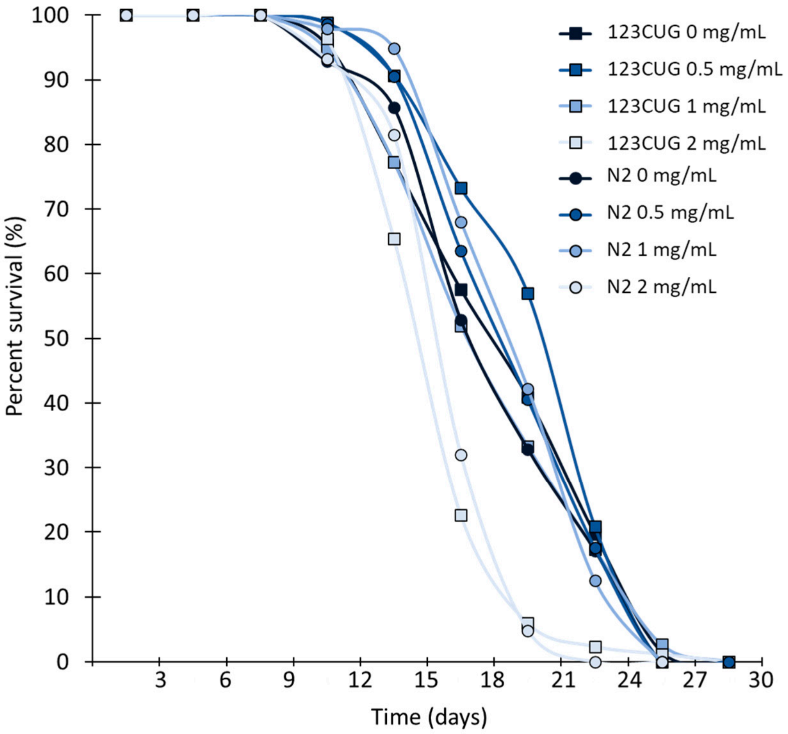

Group 1	Group 2	P-value
N2 0	123 0	0.37
N2 0	N2 0.5	0.4
N2 0	N2 1	0.1
N2 0	N2 2	0.00213
N2 0.5	123 0.5	0.053
N2 1	123 1	0.38
N2 2	123 2	0.16
123 0	123 0.5	0.16
123 0	123 1	0.61
123 0	123 2	<0.001

Figure S1. Lifespan of N2 and 123CUG following treatment with increasing doses of ATA.

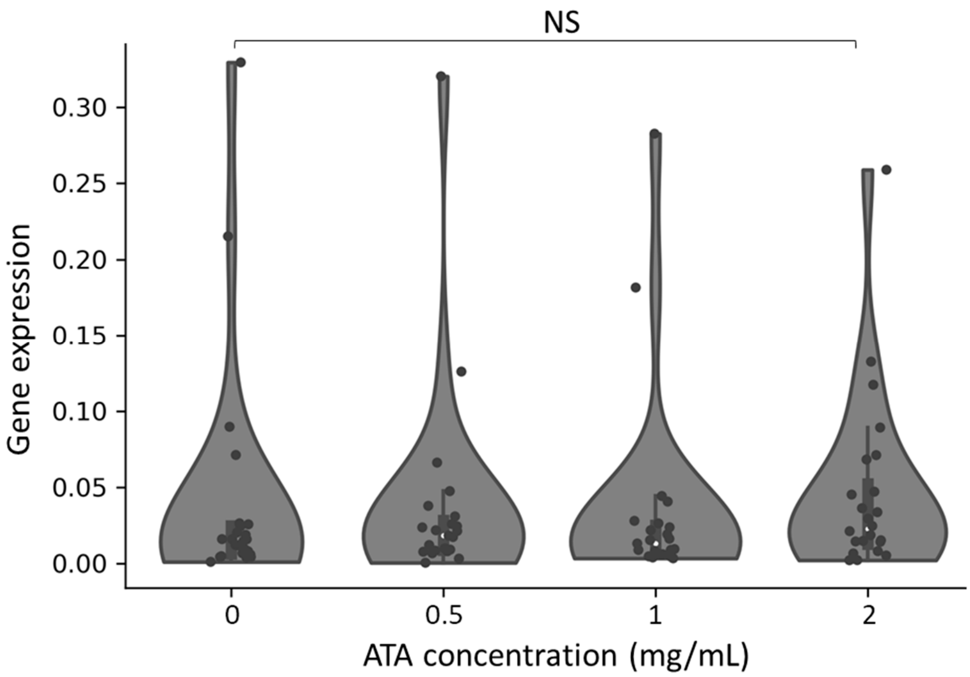

Figure S2. Gene expression of CTG-bearing genes in N2 nematodes following treatment with increasing doses of ATA. The qPCR is an average of three biological experiments and three technical replicates.

Table S1. Primer sequences for RT-qPCR.

Gene		
**ast-1**	Forward Primer	5′-AGTGGCTCGAAAATGGGGAG-3′
	Reverse Primer	5′-AGCCTGTGCGATTCCTTGAA-3′
**rga-4**	Forward Primer	5′-ACACTCTTGGAGAGGTATGCT-3′
	Reverse Primer	5′-TTCCTTGCGGAAAATCCCGT-3′
**Y75B8A.8**	Forward Primer	5′-AACATTCCCACAGGACTCGG-3′
	Reverse Primer	5′-GAAGATTGCTCTGGGGACGA-3′
**ssl-1**	Forward Primer	5′-AGCCAGGATGCGAAGATTGA-3′
	Reverse Primer	5′-GTTCGATTTTGAGCCGTTCG-3′
**R08C7.11**	Forward Primer	5′-ATGGTCGGAGGGTGTCTTCT-3′
	Reverse Primer	5′-GTCTGCGTCGTGTACTCCTT-3′
**Y23H5A.8**	Forward Primer	5′-TCGTGTGGAACGACCATCTG-3′
	Reverse Primer	5′-ATCCTGGTCCCTTGGTCCAT-3′
**mex-1**	Forward Primer	5′-AAAACCAGAGAACATTCGCACC-3′
	Reverse Primer	5′-CGACGAGCAGTGTATCCTCC-3′
**pqn-41**	Forward Primer	5′-CGCATACCAGTGACACCGAA-3′
	Reverse Primer	5′-CGGAATTCCTCATCCCTCCG-3′
**pqn-65**	Forward Primer	5′-CGACGAATCGGCTGAAGTACA-3′
	Reverse Primer	5′-GTCCTGCGACTGCTCCTAAG-3′
**Y105E8A.2**	Forward Primer	5′-GACTTATCAGGTTTGCCACGG-3′
	Reverse Primer	5′-CATTTTCGATTCGGCGGGTC-3′
**daf-2**	Forward Primer	5′-AATGCCGAGAGACACGATGC-3′
	Reverse Primer	5′-GGCTTCTTTCCACCGAGAGT-3′
**Y53G8AR.9**	Forward Primer	5′-TTTATCATCCGTCCGAGGCG-3′
	Reverse Primer	5′-CTGGCACCCTCGATTCTGAT-3′
**Y92H12A.5**	Forward Primer	5′-TCTGTTTCGAACGGGCTCTC-3′
	Reverse Primer	5′-GGCGCTTCTCATTACCCCAA-3′
**F09C8.2**	Forward Primer	5′-AATCTGAAACCGGAGCAGCA-3′
	Reverse Primer	5′-AGTTTGGTGTGAGCAGAGGAG-3′
**Y61A9LA.3**	Forward Primer	5′-GCTCTGGAGACTGGCTACAA-3′
	Reverse Primer	5′-CTTTGATCTCCTGGTCCCGC-3′
**C41D11.3**	Forward Primer	5′-TGGACAAGGCAGTTCGTTGG-3′
	Reverse Primer	5′-GTGTTGAGCACGACTTCTCG-3′
**K10D6.4**	Forward Primer	5′-TTCACCGAATGCACACCGTA-3′
	Reverse Primer	5′-CTTCCGTGGGACACACAAGA-3′
**scm-1**	Forward Primer	5′-ACCAACCACTCATCAGTCAAC-3′
	Reverse Primer	5′-ACCTGCTGCACTTCTCTGTC-3′
**M01E5.3**	Forward Primer	5′-TATGTATGCTGCTGGGCTGG-3′
	Reverse Primer	5′-ATTCACGGACGTGCACAATG-3′
**tra-1**	Forward Primer	5′-GCCCAACAAGTGTGAGTATCC-3′
	Reverse Primer	5′-TTCTCCGGTGGGTTTTCAGG-3′
**sfa-1**	Forward Primer	5′-ATTGCTGAAGCCACTGCTCT-3′
	Reverse Primer	5′-GAGCTCCGTTTGCCAAATCC-3′
**pde-6**	Forward Primer	5′-ATCGCAAATCGCCAAAGACG-3′
	Reverse Primer	5′-ACGCGTATTCCACGTCACAT-3′
**Y54G2A.3**	Forward Primer	5′-AAAAACCAGCGGATGAGGTGA-3′
	Reverse Primer	5′-TTTGAGAGCTTCTAGTTTGTACGGA-3′

**Y56A3A.6**	Forward Primer	5′-TGTTATGCCACCGCGAAAAG-3′
	Reverse Primer	5′-GAGGTGGCTGATGGTGGAAA-3′
**cdc-42**	Forward Primer	5′-CTGCTGGACAGGAAGATTACG-3′
	Reverse Primer	5′-CTCGGACATTCTCGAATGAAG-3′
**rpl-32**	Forward Primer	5′-AGGGAATTGATAACCGTGTCCGCA-3′
	Reverse Primer	5′-TGTAGGACTGCATGAGGAGCATGT-3′
